# Voltammetric methods for electrochemical characterization and quantification of artemether-based antimalarials†

**DOI:** 10.1039/d3ay01837g

**Published:** 2023-12-13

**Authors:** Anna Hildebrand, Mariam Merchant, Danny O'Hare

**Affiliations:** a Department of Bioengineering, Imperial College London London SW7 2AZ UK a.hildebrand21@imperial.ac.uk mariam.merchant19@imperial.ac.uk d.ohare@imperial.ac.uk

## Abstract

Every year substandard and falsified (SF) artemisinin derivative-based antimalarials are responsible for the loss of 450 000 deaths and billions of GBP. The lack of infrastructure and funds to support pharmaceutical quality control in many low-and-middle-income countries contributes to this problem. This work assesses fitness for purpose of voltammetric methods for identification and quantification of artemether in the presence of excipients. Electrochemical characterization of artemether using cyclic voltammetry shows that the reduction of artemether is chemically irreversible within the potential range of −0.4 V to −1.4 V. A chronocoulometric quantification algorithm for artemether is created and tested with pure artemether, as well as filtered and unfiltered Riamet® tablets. Filtration of Riamet® tablets provides no additional benefit for the quantification of artemether in Riamet®. In addition, artemether's response to pH indicates possible protonation and coupled homogeneous chemistry. Finally, sodium sulfite is an effective means of removing dissolved oxygen and improving artemether signal resolution in air-equilibrated PBS. This concludes that electrochemical analysis is a promising method for artemether identification and quantification.

## Abstract 2

Electrochemical characterization and quantification of artemether established with cyclic voltammetry and chronocoulometry. Fitness for purpose validated for use of voltametric methods for analysis of artemether in the presence of excipients.

## Graphical abstract

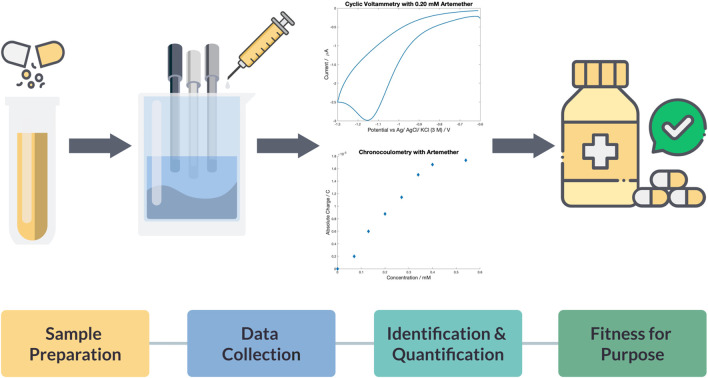

## 1. Introduction

Artemisinin-based combination therapy (ACTs) are the first line of treatment for *P. falciparum* malaria,^2^ but substandard and falsified (SF) ACTs pose a significant threat to malaria control.^3^ It has been estimated that falsified antimalarials contribute to nearly 450 000 preventable deaths every year.^1^ The mean prevalence of substandard and falsified antimalarials in low-and-middle-income countries (LMIC) is 19.1%.^4^

A well-equipped medicines quality control laboratory (MQCL) integrated in the pharmaceutical supply chain is a vital component of any medicine quality assurance system.^5^ However, the gold standard for pharmaceutical quality analysis is high performance liquid chromatography (HPLC), which is inaccessible for many LMIC due to lack of infrastructure and funds.^5^ There are several technologies that have attempted to address this need. Spectroscopic devices are highly accurate, but they can be complex to use and are too expensive for LMIC (∼£50 000 per unit).^6^ Other less costly methods are often only qualitative or semi-quantitative, in turn preventing the quantification of the active pharmaceutical ingredient (API), which is vital to distinguish authentic from substandard medicines with reduced API.^7^ Thus, there is a need for a low-cost (less than £1000), field-based device for artemisinin derivative medicine quality screening.

The research presented in this work will be used to address the need for the detection of substandard and falsified artemisinin-based antimalarials through the development of a medicine quality screening device. This work specifically explores the feasibility of voltammetric methods for the basis of the device technology.

The detection of artemisinin derivatives with voltammetry utilizes the unique electrochemical properties of artemisinin derivatives. All artemisinin-based compounds contain an endoperoxide moiety, which is vital for their function. *In vivo*, heme catalyses the reductive cleavage of the endoperoxide moiety from artemisinin, which results in the production of a reactive species that targets malaria-specific proteins for elimination.^8–10^ Thus, cyclic voltammetry was used to investigate overall electrochemical characteristics and obtain semi-quantitative mechanistic data. Quantification was then undertaken with chronocoulometry.

We have evaluated our algorithm with both pure artemether and Riamet® 20 mg/120 mg tablets, a pharmaceutical artemether–lumefantrine formulation. A syringe-filter apparatus was assembled to compare unfiltered Riamet® and filtered Riamet® to pure artemether. Both chronocoulometric calibration curves and cyclic voltammograms were compared.

In addition to the evaluation of the voltammetric signal from pure artemether and artemether in the presence of excipients, the electrochemical properties of artemether were investigated. This investigation was conducted with cyclic voltammetry by determining artemether's response to scan rate and buffer pH changes. An analyte's response to scan rate can provide kinetic and mechanistic information.^11^ Shifts in redox half-peak potential and current in response to pH changes can indicate the possible protonation or deprotonation of the analyte, which can provide evidence for coupled homogeneous chemistry.^12^

Finally, this work investigates the possibility of signal interference from dissolved oxygen. This is a concern as the reduction potential of oxygen on glassy carbon electrodes (−0.6 V *vs.* Ag/AgCl^13^) occurs near the reduction potential of artemether (−1.2 V *vs.* Ag/AgCl) in phosphate buffer with a pH of 7.55. Ultimately this technology will be implemented in the field where temperature, pressure and humidity will not be controlled, all of which cause the concentration of dissolved oxygen to vary (from 92 μM to 375 μM) (ESI 1.0†) Thus, the effect of dissolved oxygen on artemether was studied using cyclic voltammetry. In addition, cyclic voltammetry and chronocoulometry were used to investigate if dissolved oxygen removal with sodium sulfite can allow for artemether signal resolution.

To date, cyclic voltammetry is the only voltammetric method used to study artemisinin derivatives that has been documented.^14–16^ Obwayo *et al.*, Jain *et al.*, and Zhang *et al.* report that the reduction of artemisinin derivatives on glassy carbon is chemically irreversible from 0 V to −1.5 V, with a reduction potential around −1.0 V *vs.* Ag/AgCl.^14–16^ In addition, Zhang *et al.* found evidence for adsorption of artemisinin at the electrode surface.^15^

The objective of this work is to evaluate feasibility of voltammetry for the detection and quantification of artemether. Discrepancies between pure artemether and artemether in the presence of excipients were identified. Kinetic and mechanistic properties of artemether were also investigated.

## 2. Materials and methods

### 2.1 Electrochemical cell components

All experiments were performed using a 1.6 mm glassy carbon working electrode (IJ Cambria Scientific), an Ag/AgCl/KCl (3 M) reference electrode (RE-B, IJ Cambria Scientific) and a Pt wire auxiliary electrode. A 5 cm platinum wire auxiliary electrode (IJ Cambria Scientific) was used for experiments with Riamet® (Fig. 3 and 4) and experiments with nitrogen-, air-, and oxygen-equilibrated PBS (Fig. 7). A 23 cm platinum coiled wire auxiliary electrode (IJ Cambria Scientific) was used for experiments with artemether's response to scan rate (Fig. 5), buffer pH changes (Fig. 6), and sodium sulfite (Fig. 8 and 9). There was evidence that artemether adsorbs on the electrode surface when recording subsequent measurements, so a clean electrode was used for each measurement.

The supporting electrolyte used in all experiments was PBS with a neutral pH with 0.01 M phosphate buffer, 0.0027 potassium chloride and 0.137 sodium chloride (ESI 2.1†) (Sigma-Aldrich). The volume of PBS used for all experiments was 14 mL. For all experiments except the investigation into the effect of dissolved oxygen on artemether and the effect of sodium sulfite on artemether, the PBS solution was degassed with nitrogen gas (BOC) for 20 minutes prior to the addition of the analyte. The solution was blanketed with nitrogen during recordings and bubbled with nitrogen for 30 seconds between recordings.

Dimethyl sulfoxide (DMSO) (Sigma-Aldrich) was selected as the extraction solvent for all experiments.

### 2.2 Electrode preparation

Glassy carbon electrodes were abraded in 1 μm, 0.3 μm and 0.05 μm alumina slurry (Buehler) on polishing pads of grade 1 μm, 0.3 μm and 0.05 μm (Buehler). The electrodes were then sonicated in 20 mL of 1 : 1 solution of deionized water and acetone (VWR).

### 2.3 Data collection and analysis

Ivium CompactStat and IviumSoft were utilized for data collection. Data visualization and data analysis were executed with Matlab 2022b.

### 2.4 Development of chronocoulometric algorithm

**Fig. 1:**
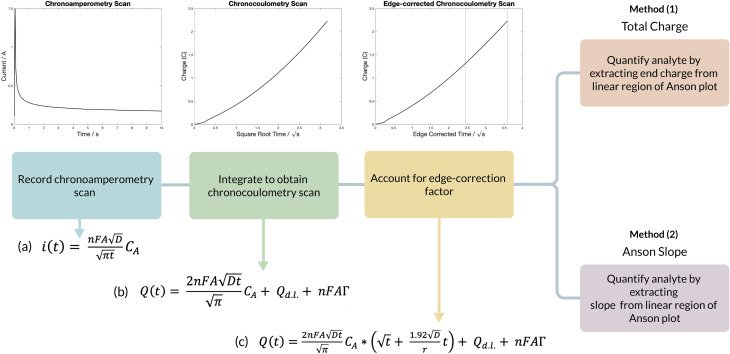
Algorithm for artemether quantification using chronocoulometry. Variables: *n* is the number of electrons transferred in the redox event, *A* is the area of the electrode (m^2^), *D* is the diffusion coefficient of the analyte (m^2^ s^−1^), *C*_A_ is the initial concentration of the analyte (mol m^−3^), *r* is the radius of the electrode (m^2^), *Q*_d.l._ is the double layer charge, and *nFAΓ* is the adsorbed species (*Γ* is the amount of adsorbed reactant (moles cm^−2^)).

The chronocoulometric algorithm that was developed to quantify artemether is outlined in Fig. 1. For chronoamperometric scans, a Heaviside step function is applied and the resulting current then decays to zero,^17^ as described by the Cottrell equation Fig. 1 (equation a). Integrating the Cottrell equation can isolate the double layer charge (*Q*_d.l._) and charge arising from the adsorbed drug and its intermediates (*nFAΓ*) from the faradaic current to increase the signal to noise ratio.^18^ This relationship is described by the Anson equation Fig. 1 (equation b). At long times, however, diffusion at the electrode surface is no longer strictly planar, as the spherical growth of the diffusion layer permits radial diffusion to the electrode edge.^19–22^ Thus, the Anson equation can be modified with a correction factor to account for radial diffusion Fig. 1 (equation c). In the absence of adsorption, the absorbed constant is zero and quantification of the drug from the edge-corrected Anson equation can be obtained from total charge by correcting for the double-layer charge that is attributed to the blank solution. However, in the event of adsorption, it is difficult to isolate the adsorbed charge from the total charge, so quantification of the drug is obtained by extracting the diffusion-limited current from the edge-corrected Anson equation.

Evidence of adsorption of artemether at the electrode surface was present, so the two quantification methods were tested by Fig. 1 (method 1) extracting the end charge value from the Anson plot and Fig. 1 (method 2) extracting the slope from the linear region of each Anson plot. These two metrics were compared with pure artemether, filtered Riamet® tablets and unfiltered Riamet® tablets. Additionally, comparison of quantification from total charge with artemether in nitrogen-equilibrated PBS and artemether in 1 mM sodium sulfite with air-equilibrated PBS was conducted.

### 2.5 Comparison of pure artemether, filtered Riamet® tablets and unfiltered Riamet® tablets with cyclic voltammetry and chroncoulometry

**Fig. 2:**
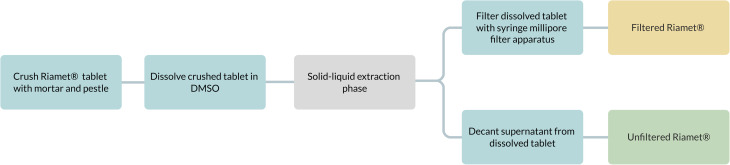
Protocol for sample preparation of filtered and unfiltered Riamet® tablets.

For the pure API preparation, pure artemether (Cayman Chemical) was dissolved in DMSO. Two API extraction methods were compared: unfiltered Riamet® and filtered Riamet®. First, a Riamet® tablet was crushed with a mortar and dissolved in DMSO. For the unfiltered method, the DMSO/Riamet® solution was decanted to isolate the supernatant solution containing the dissolved drug from excipient solids. For the filtered method, the DMSO/Riamet® solution was filtered with a 0.22 μm Millipore filter (Merck) and syringe apparatus (Merck) (ESI 2.2†). The artemether concentration in the DMSO stock solution was 16.76 mM. Sample preparation methods for Riamet® are shown in Fig. 2.

Chronocoulometric calibration curves were created with standard additions of aliquots of [0 μL, 84 μL, 198 μL, 171 μL, 230 μL, 640 μL, 480 μL] of the artemether/DMSO stock solution to 14 mL of nitrogen-equilibrated PBS pH of 7.48 for final artemether concentrations of [0 mM, 0.07 mM, 0.13 mM, 0.20 mM, 0.27 mM, 0.34 mM, 0.40 mM]. A single potential step was applied with an *E*_o_ of −0.6 V for 5 seconds, and an *E*_1_ of −1.1 V for 10 seconds. The interval time was 0.05 seconds, and the current range was 100 μA. Data for quantification was taken from 5 to 10 seconds after the potential step to −1.1 V.

Cyclic voltammograms were recorded at 0.27 mM artemether from with pure artemether, filtered Riamet® and unfiltered Riamet®. The potential was cycled from −0.6 V to −1.3 V with a scan rate of 100 mV s^−1^. An *E*_step_ of 10 mV, current range of 100 μA, equilibrium time of 0 s, 10 Hz filter and high stability setting were used.

### 2.6 Cyclic voltammetry with artemether and variable scan rates

430 μL of 16.76 mM artemether/DMSO stock solution was added to 14 mL of nitrogen-equilibrated PBS with pH of 7.54 for a final artemether concentration of 0.20 mM. Cyclic voltammetry scans were recorded with scan rates of [50 mV s^−1^, 100 mV s^−1^, 200 mV s^−1^, 300 mV s^−1^, 400 mV s^−1^, 500 mV s^−1^]. The potential was cycled from −0.6 V to −1.3 V. An *E*_step_ of 10 mV, current range of 100 μA, equilibrium time of 0 s, 10 Hz filter and high stability setting were used.

### 2.7 Artemether's response to pH with cyclic voltammetry

Three PBS solutions were prepared with a pH of 6.45, 7.54 and 8.47. 430 μL of 16.76 mM artemether/DMSO stock solution was added to 14 mL of nitrogen-equilibrated PBS for a final artemether concentration of 0.20 mM. Cyclic voltammograms were recorded and the potential was cycled from −0.6 V to −1.1 V with a scan rate of 100 mV s^−1^. An *E*_step_ of 10 mV, current range of 100 μA, equilibrium time of 0 s, 10 Hz filter and high stability setting were used.

### 2.8 Dissolved oxygen effect on artemether with cyclic voltammetry

PBS with pH of 7.55 was equilibrated with nitrogen, air, and oxygen. PBS was degassed for 20 minutes with nitrogen (BOC) or oxygen (BOC) for measurements with either gas. The gases were moistened by bubbling through the electrolyte solution to prevent evaporative cooling. The electrolyte solution remained in ambient conditions for measurements with air-equilibrated PBS. 430 μL of 16.76 mM artemether/DMSO stock solution was added to 14 mL of each gas-equilibrated PBS for a final artemether concentration of 0.20 mM. Cyclic voltammograms were recorded and the potential was cycled from −0.6 V to −1.1 V with a scan rate of 100 mV s^−1^. An *E*_step_ of 10 mV, current range of 100 μA, equilibrium time of 0 s, 10 Hz filter and high stability setting were used.

### 2.9 Comparison of artemether in air-equilibrated PBS with sodium sulfite and nitrogen-equilibrated PBS with cyclic voltammetry and chronocoulometry

14 mL PBS with 1 mM sodium sulfite (Sigma-Aldrich) and pH of 7.55 was prepared and left under ambient conditions. 14 mL PBS with pH of 7.54 was degassed with nitrogen.

For the chronocoulometric calibration curve, standard additions of [0 μL, 84 μL, 198 μL, 171 μL, 230 μL, 640 μL, 480 μL] of the 16.76 mM artemether/DMSO stock solution were added to each PBS for final artemether concentrations of [0 mM, 0.07 mM, 0.13 mM, 0.20 mM, 0.27 mM, 0.34 mM, 0.40]. A single potential step was applied with an *E*_o_ of −0.6 V for 5 seconds, and an *E*_1_ of −1.1 V for 10 seconds. The interval time was 0.05 seconds and the current range was 100 μA. Data for quantification was taken from 5 to 10 seconds after the potential step to −1.1 V.

Cyclic voltammograms were recorded with 0.20 mM artemether in each PBS. The potential was cycled from −0.6 V to −1.3 V with a scan rate of 100 mV s^−1^. An *E*_step_ of 10 mV, current range of 100 μA, equilibrium time of 0 s, 10 Hz filter and high stability setting were used.

## 3. Results and discussion

### 3.1 Electrochemical characterization of pure artemether, filtered and unfiltered Riamet® tablets with cyclic voltammetry

**Fig. 3:**
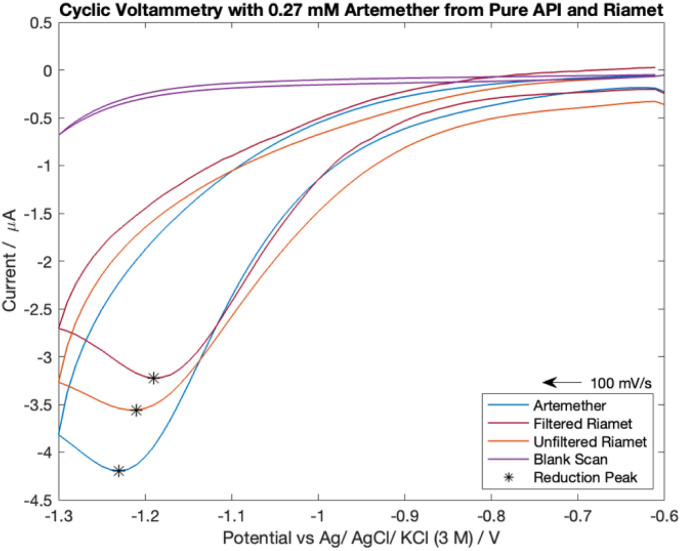
Cyclic voltammetry scans from 0.27 mM of pure artemether, filtered Riamet® and unfiltered Riamet® in nitrogen-equilibrated PBS (pH of 7.48).

The cyclic voltammograms for the comparison of 0.27 mM pure artemether with filtered and unfiltered Riamet® tablets can be seen in Fig. 3. The scans with filtered and unfiltered Riamet® exhibit similar characteristics as the scan with pure artemether. Namely, all scans exhibit chemical irreversibility within −0.6 V to −1.3 V with a single reduction peak around −1.2 V to −1.25 V. The reduction peak potential is most negative for pure artemether (−1.23 V), then unfiltered Riamet® (−1.21 V) and finally filtered Riamet® (−1.19 V). The negative shift in reduction peak potential from filtered Riamet® to unfiltered Riamet® could be due to the loss of acidic components when filtering. Specifically, the filtration of hypromellose (pH of 5.5 to 8 in water^23^) and croscarmellose sodium (pH of 5 to 8.2 in water^24^) may result in an increase of pH with the filtered Riamet®. The reduction potential from artemether, in its pure form as well as from filtered and unfiltered Riamet® tablets, shifts negatively with increasing concentration. In addition to the shift in reduction peak potential, the reduction peak current amplitude is the largest for pure artemether (−3.54 μA), then unfiltered Riamet (−1.21 μA) and finally filtered Riamet® (−1.19 μA). The difference in reduction peak current is likely due to losses of artemether during extraction and filtration.

### 3.2 Quantification of pure artemether, filtered and unfiltered Riamet® tablets with chronocoulometry

**Fig. 4:**
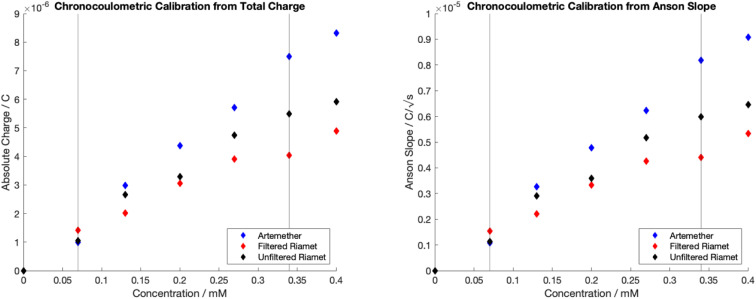
Chronocoulometric calibration curves with pure artemether, filtered Riamet® and unfiltered Riamet® in nitrogen-equilibrated PBS (pH of 7.48). (Left) Obtained by extracting total charge (right) Obtained by extracting Anson slope.

Chronocoulometric calibration curves for quantification of artemether, filtered Riamet® and unfiltered Riamet® are visualized in Fig. 4. Adsorption results in measurable carry-over, therefore electrodes were freshly cleaned (section 2.2) for each data point in Fig. 4. Linear regression models of the dynamic range (0.07 mM to 0.34 mM) for calibrations with total charge and Anson slope were created (Table S2†). Regression models for artemether, filtered Riamet® and unfiltered Riamet® for total charge and Anson slope are statistically significant (*p*-value < 0.05, *n* = 5, total charge *p* = [0.00038, 0.00408, 0.00168], Anson slope *p* = [0.00037, 0.00413, 0.00165]) (Table 1). In addition, there is no evidence that the intercept for any regression model is significantly different from zero (*p*-value < 0.05, *n* = 5, total charge *p* = [0.31233, 0.08129, 0.59204], Anson slope *p* = [0.31093, 0.07928, 0.59370]) (Table 1), indicating that adsorption at the electrode surface does not significantly affect the data. The slope of each regression model is also significantly different from zero (*p*-value < 0.05, *n* = 5, total charge *p* = [0.00038, 0.00408, 0.00168], Anson slope *p* = [0.00037, 0.00413, 0.00165]) (Table 1), which can be interpreted as non-zero sensitivity. In addition, using a scale factor of 7, the dynamic range of the calibration curve (0.07 mM to 0.34 mM) correlates to quantification of artemether from 2.94 mg to 28.1 mg, with the limit of quantification being 2.94 mg artemether. In relation to Riamet® (containing 20 mg of artemether), this corresponds to a linear detection range from 14.7% API to 140.5% API. Thus, these methods can accurately detect Riamet® samples containing less than 85% artemether and more than 115% artemether, allowing for identification of ‘out of specification’^25^ (substandard) samples.

**Table 1:** Resulting *p*-values from linear regression models of dynamic range for artemether, filtered Riamet® and unfiltered Riamet® for chronocoulometric calibrations from total charge and Anson slope

Linear regression model *p*-values			
	Artemether	Filtered Riamet®	Unfiltered Riamet®
**Total charge (*n* = 5 for all groups)**			
Intercept	0.31233	0.08129	0.59204
Slope	0.00038	0.00408	0.00168
Model	0.00038	0.00408	0.00168

**Anson slope (*n* = 5 for all groups)**			
Intercept	0.31093	0.07928	0.59370
Slope	0.00037	0.00413	0.00165
Model	0.00037	0.00413	0.00165

*t*-Tests were conducted on pairwise groups to determine if there is significant difference in mean sensitivities between calibrations with artemether, filtered Riamet® and unfiltered Riamet® (Table S4†). There is a significant difference in sensitivities at the 5% significance level with calibrations with total charge between pairs of artemether and filtered Riamet® (*n* = 5, *p* = 6.61 × 10^−5^), artemether and unfiltered Riamet® (*n* = 5, *p* = 0.0036), and filtered Riamet® and unfiltered Riamet® (*n* = 5, *p* = 0.0114). Additionally, there is also a significant difference with calibrations from Anson slope between pairs of artemether and filtered Riamet® (*n* = 5, *p* = 6.37 × 10^−5^), artemether and unfiltered Riamet® (*n* = 5, *p* = 0.0036), and filtered Riamet® and unfiltered Riamet® (*n* = 5, *p* = 0.0109) at the 5% significance level.

*F*-tests on each calibration method were conducted to determine if there is any significant difference in variability between calibrations for artemether, filtered Riamet® and unfiltered Riamet® (Table S3†). There is no significant difference in mean sensitivity variance observed for the calibration with total charge between the three sample groups (*F*-stat of 2.136 < 3.739 of *F*-critical, *n* = 15 for within sample variance, *n* = 3 for between sample variance). However, the calibration with Anson slope yields significantly different variance with sensitivities between the three groups (*F*-stat of 4.3273 > 3.739 of *F*-critical, *n* = 15 for within sample variance, *n* = 3 for between sample variance).

While there is a significant difference in mean sensitivity between total charge calibrations pairs of artemether and filtered Riamet®, and artemether and unfiltered Riamet®, there is no significant difference in mean sensitivity variance between artemether and both filtered/unfiltered Riamet®. Thus, as mean sensitivity variance is equal, a correction factor can be applied to account for the signal differences between artemether and filtered/unfiltered Riamet®. The artemether mean recovery rate is 63.54% for filtered Riamet® (*n* = 5, standard deviation of 7.00%) and 83.11% for unfiltered Riamet® (*n* = 5, standard deviation of 9.24%).

In summary, there is a significant difference in mean sensitivity between artemether, filtered Riamet® and unfiltered Riamet® for both calibrations with total charge and Anson slope. However, quantification with total charge is more precise as there is no significant difference in mean sensitivity variance between the calibration from artemether, filtered Riamet® and unfiltered Riamet®. Adsorption is not significant for quantification with either total charge or Anson slope. Finally, there is no evidence that filtration improves analyte signal as there is no significant difference in mean sensitivity variance between filtered and unfiltered Riamet® tablets when quantifying with total charge.

### 3.3 Cyclic voltammetry with artemether and variable scan rates

**Fig. 5:**
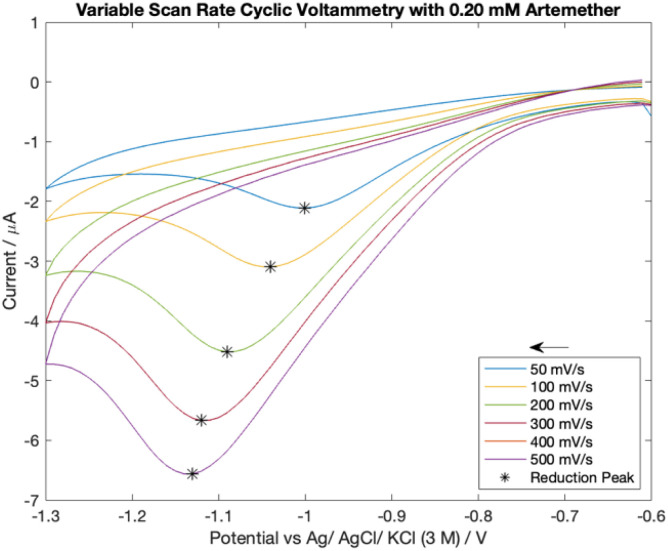
Cyclic voltammetry scans with 0.20 mM artemether in nitrogen-equilibrated PBS (pH of 7.54). Scan rates vary from 50 mV s^−1^ to 500 mV s^−1^.

Cyclic voltammetry scans with 0.20 mM artemether in nitrogen-equilibrated PBS with scan rates from 50 mV s^−1^ to 500 mV s^−1^ can be seen in Fig. 5. Reduction peak potential becomes more negative with increasing scan rates, from −1.0 V at 50 mV s^−1^ to −1.13 V at 500 mV s^−1^. In addition, the absence of an oxidation peak despite increasing scan rate indicates that the reduction of artemether is chemically irreversible within −0.6 V to −1.3 V. From 50 mV s^−1^ to 500 mV s^−1^, there is a linear relationship between reduction peak current density and square root of the scan rate as well as the reduction peak potential and log of the scan rate (Fig. S2†).

The reduction peak from artemether plateaus at scan rates above 500 mV s^−1^. Beyond scan rates 500 mV s^−1^, artemether begins to exhibit unusual behaviour (Fig. S1†). Interestingly, a second reduction peak occurs around −1 V at scan rates above 500 mV s^−1^, and this second peak (*E*_p_ of −1.01 V and *i*_p_ of−5.48 μA at 900 mV s^−1^) overwhelms the signal from artemether so that the reduction peak from artemether is no longer distinct. This is not amenable to routine mechanistic attribution and beyond the scope of this paper.

### 3.4 Artemether's response to pH with cyclic voltammetry

Analyte response to pH was investigated with cyclic voltammetry as literature reports that pH has a direct effect on artemisinin derivatives' reduction peak potential.^16,26^ The pH of the supporting electrolyte ranged from pH of 6.5 to 8.5. The p*K*_a_ of artemether is 3.48 (ref. 27) so highly acidic media was avoided to prevent signal interference. In addition, highly alkaline media was avoided as it can damage carbon electrodes^28^ and is hazardous to work with.

**Fig. 6:**
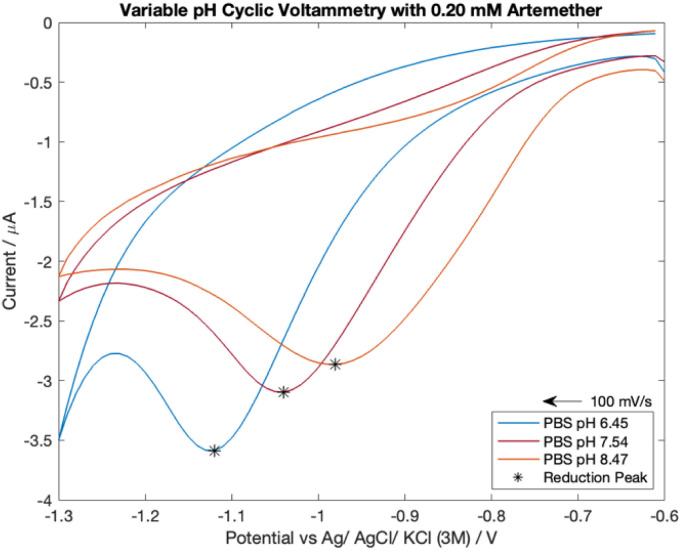
Cyclic voltammetry scans of 0.20 mM artemether in nitrogen-equilibrated PBS (pH of 6.45, 7.54 and 8.47).

Cyclic voltammograms for 0.20 mM artemether in nitrogen-equilibrated PBS with pH of 6.45, 7.54 and 8.47 are shown in Fig. 6. Cyclic voltammetry reveals that half-peak current amplitude increases as media becomes more acidic (from −2.12 μA to −2.45 μA to −3.27 μA from pH of 8.47 to 7.54 to 6.45), indicating that protonation of artemether or its reaction intermediates may be mechanistically significant. Additionally, the reduction potential of artemether decreases as media becomes more alkaline, from −0.98 V with a pH of 8.47 to −1.05 V with a pH of 7.54 to −1.12 V with a pH of 6.45.

Examination of the shift of half-peak potentials with respect to pH reveals there is a 100 mV shift from pH of 6.45 (−0.98 V) to 7.54 (−0.88 V) and an 80 mV shift from pH 7.54 to 8.47 (−0.80 V). Thus, there is a 92 mV shift in half-peak potential per unit pH from a pH of 6.5 to 7.5, and an 86 mV shift in half-peak potential per unit pH from a pH of 7.5 to 8.5. A shift in reduction half-peak potential may indicate possible protonation of the artemisinin derivatives or their reaction intermediates, which can provide evidence for coupled homogeneous chemistry.^12^ Therefore, if the reduction of artemether is coupled with protonation, we would expect a ∼60 mV shift in reduction half-peak potential per unit pH (ESI 3.3†). Although the shifts in half-peak potentials per unit pH are not equal to the theoretical shift in half-peak potential that indicates a dual electron transfer coupled with protonation, there is a direct relationship between reduction half-peak potential and pH within a neutral pH range.

Voltammetric data is consistent with protonation of reaction intermediates. Artemether responds to an increase in phosphate buffer pH with a positive shift in reduction potential (and therefore half-peak potential) as well as a decrease in reduction peak current amplitude. Surprisingly, despite remaining more than 3 pH units above the p*K*_a_ of artemether, artemether was highly respondent to pH changes within the neutral pH range. This indicates that pH control is vital for accurate artemether analysis with voltammetry.

### 3.5 Dissolved oxygen effect on artemether with cyclic voltammetry

**Fig. 7:**
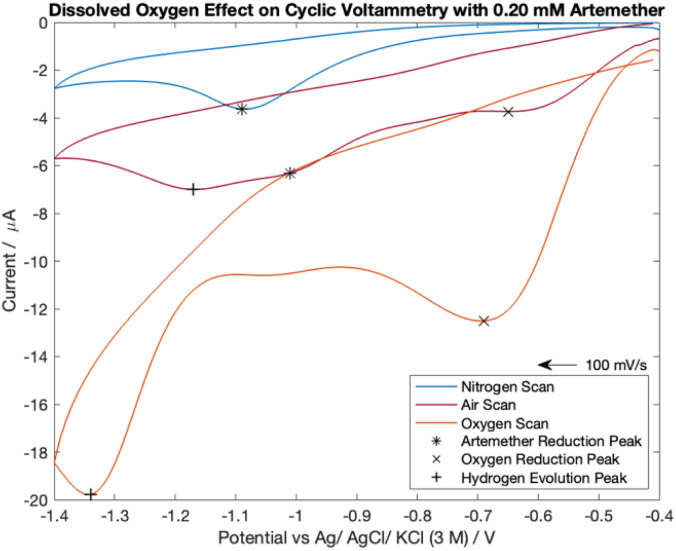
Cyclic voltammetry scans comparing 0.20 mM of artemether in nitrogen-, oxygen- and air-equilibrated PBS (pH of 7.55).

The cyclic voltammetry scans for artemether in nitrogen-, oxygen- and air-equilibrated PBS are visualized in Fig. 7. Notably, all scans lack an oxidation peak and are chemically irreversible within −0.4 V to −1.4 V. The nitrogen-equilibrated scan reveals the isolated response of artemether, with a reduction potential of −1.09 V. A peak from oxygen reduction is present in the scans with PBS equilibrated in air (−0.65 V) and oxygen (−0.69 V). The presence of oxygen causes the reduction potential of artemether to shift towards (positive) the reduction potential for oxygen. This occurrence is clearly seen in the scan under air (−1.01 V). The signal from artemether in oxygen-equilibrated PBS is overwhelmed by the signal from oxygen so the peak from artemether is no longer distinct. An additional peak is present in the scans with PBS equilibrated in air (−1.17 V) and oxygen (−1.34 V) at more negative potentials, representing hydrogen evolution. These results indicate that control of oxygen is necessary for accurate detection of artemether.

Cyclic voltammograms comparing artemether in air-equilibrated PBS with the sum of oxygen-equilibrated PBS and artemether in nitrogen-equilibrated PBS show discrepancies in peak height and intensity (Fig. S3†). This suggests pH changes induced by oxygen reduction directly affect the signal from artemether.

### 3.6 Sodium sulfite in air-equilibrated PBS effect on artemether with cyclic voltammetry

Dissolved oxygen is reducible to hydrogen peroxide in the potential range examined. Therefore, 1 mM sodium sulfite was added to air-equilibrated PBS to determine if sodium sulfite can effectively be used for oxygen removal in field-based settings. As shown in blank cyclic voltammetry scans, the addition of 1 mM sodium sulfite to air equilibrated PBS effectively removes the reduction peak from dissolved oxygen at −0.60 V (Fig. S5†).

**Fig. 8:**
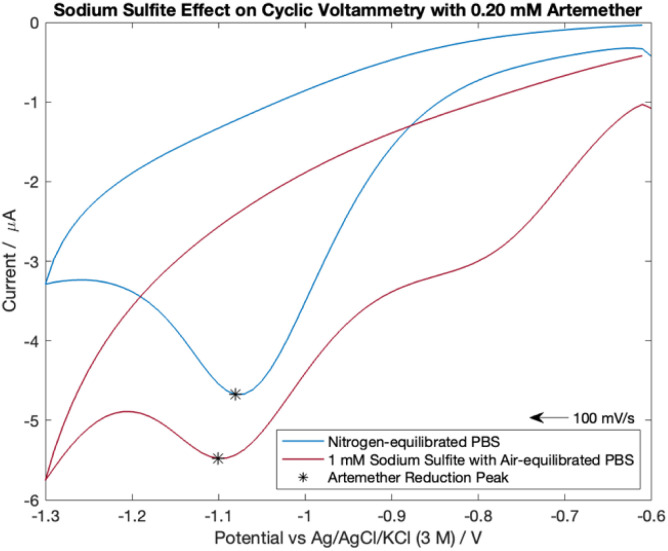
Cyclic voltammetry scans comparing 0.20 mM of artemether in nitrogen-equilibrated PBS (pH of 7.54) and 1 mM sodium sulfite in air-equilibrated PBS (pH of 7.55).

Cyclic voltammograms for 0.20 mM artemether in nitrogen-equilibrated PBS and air-equilibrated PBS with 1 mM sodium sulfite are shown in Fig. 8. As shown, cyclic voltammograms exhibit chemically irreversibility within −0.6 V to −1.3 V with reduction peak potentials of −1.10 V (air-equilibrated PBS with 1 mM sodium sulfite) and −1.08 V (nitrogen-equilibrated PBS). The reduction peak potential from artemether becomes more negative with increasing concentration for both nitrogen-equilibrated PBS and 1 mM sodium sulfite in air-equilibrated PBS (Fig. S5†). However, the artemether reduction peak current amplitude for nitrogen-equilibrated PBS (−4.06 μA) is larger than the peak for air-equilibrated PBS with 1 mM sodium sulfite (−1.40 μA). This could be because an additional reduction peak at −0.80 V makes it difficult to fully isolate the signal from artemether in air-equilibrated PBS with 1 mM sodium sulfite. Notably, the reduction peak at −0.80 V is present in all cyclic voltammograms with sodium sulfite air-equilibrated PBS with concentrations of artemether ranging from 0.07 mM to 0.54 mM (Fig. S5†). This reduction peak at −0.80 V increases in amplitude with increasing concentration of artemether until it saturates at 0.20 mM artemether (around −2 μA). Surprisingly, the reduction peak at −0.80 V is not present in blank cyclic voltammetry scans, which indicates that artemether or its reaction intermediates may interact with sodium sulfite to produce a second reduction peak at −0.80 V. Finally, hysteresis is larger for cyclic voltammetry scans with sodium sulfite air-equilibrated PBS than nitrogen-equilibrated PBS, and hysteresis is constant among all concentrations of artemether for both sodium sulfite air-equilibrated PBS and nitrogen equilibrated PBS (Fig. S5†).

The addition of 1 mM sodium sulfite improves artemether signal resolution in air-equilibrated PBS. An oxygen reduction peak at −0.80 V is still present in scans with sodium sulfite PBS, but the oxygen peak saturates at 0.20 mM artemether. Additionally, hysteresis is also constant with artemether in nitrogen-equilibrated PBS and sodium sulfite air-equilibrated PBS. Therefore, sodium sulfite could allow for reliable calibration of artemether in air-equilibrated PBS.

### 3.7 Quantification of artemether with sodium sulfite in air-equilibrated PBS with chronocoulometry

**Fig. 9:**
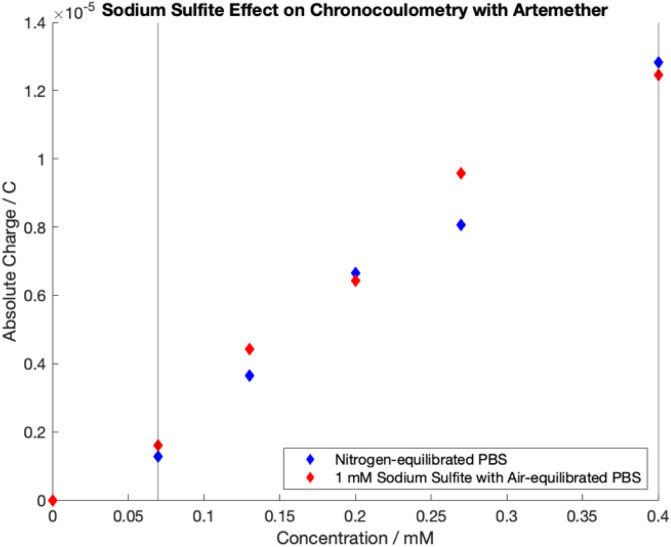
Chronocoulometric calibration curves obtained by extracting total charge from artemether in nitrogen-equilibrated PBS (pH of 7.54) and 1 mM sodium sulfite in air-equilibrated PBS (pH of 7.55).

Chronocoulometric calibration curves for artemether quantification with nitrogen-equilibrated PBS and 1 mM sodium sulfite in air-equilibrated PBS are shown in Fig. 9. Quantification was achieved through extraction of total charge from chronocoulometric plots as it was found that there is no added benefit to quantification from Anson slope (Fig. 4). Linear regression models of the dynamic range (0.07 mM to 0.40 mM) were created for both calibration curves (Table S7†). Regression models for artemether in nitrogen-equilibrated PBS (*p*-value < 0.05, *n* = 5, *p* = 0.00031) and 1 mM sodium sulfite in air-equilibrated PBS (*p*-value < 0.05, *n* = 5, *p* = 0.00115) are statistically significant. In addition, the slope of both calibration curves are also statistically significant (*p*-value < 0.05, *n* = 5, nitrogen-equilibrated PBS *p* = 0.00031, sodium sulfite air-equilibrated PBS *p* = 0.00115). There is no evidence of adsorption at the electrode surface in either model as there is no evidence that either model has a non-zero intercept (*p*-value < 0.05, *n* = 5, nitrogen-equilibrated PBS *p* = 0.14648, sodium sulfite air-equilibrated PBS *p* = 0.84383).

As revealed by *t*-tests on calibrations with artemether in nitrogen-equilibrated PBS and artemether in 1 mM sodium sulfite with air-equilibrated PBS, there is no significant difference in mean sensitivity at the 5% significance level between the two groups (*p* = 0.2970, *n* = 5). *F*-tests reveal there is a significant difference in mean sensitivity variance between calibrations in nitrogen-equilibrated PBS and 1 mM sodium sulfite in air-equilibrated PBS (*F*-stat of 104.77 > 3.36 of *F*-critical, *n* = 10 for within sample variance, *n* = 2 for between sample variance) (Table S8†).

There is no difference in mean sensitivity between artemether quantification with chroncoulometry in nitrogen-equilibrated PBS and 1 mM sodium sulfite in air-equilibrated PBS. The difference in variance between artemether quantification in the two mediums is expected as addition of another variable will increase variability. Thus, the addition of sodium sulfite to air-equilibrated PBS can allow for translation of methods to field-based settings.

## 4. Conclusions

Cyclic voltammograms reveal that artemether, filtered Riamet® and unfiltered Riamet® are chemically irreversible within the potential range of −0.6 V to −1.3 V with reduction potentials within −1.2 V to −1.25 V in PBS with a pH of 7.48. Excipients in Riamet® tablets were not found to affect the signal from artemether with both cyclic voltammetry and chronocoulometry.

Chronocoulometric calibration curves obtained from both total charge and Anson slope reveal there is a significant difference in mean sensitivity between quantification with artemether, filtered and unfiltered Riamet®. Unlike quantification with Anson slope, there is no significant difference in mean sensitivity variance between artemether, filtered and unfiltered Riamet® when quantifying with total charge. Thus, quantification with total charge is more precise and a correction factor can be applied to account for the difference in mean sensitivity between artemether and filtered/unfiltered Riamet®. It follows that filtration of Riamet® provides no added benefit. Finally, these methods can accurately quantify artemether from Riamet® samples within the 14.7% API to 140.5% API range, justifying fitness for purpose for detection of substandard drug samples.

Cyclic voltammograms with variable scan rates reveal the reduction of artemether is diffusion-controlled from 50 mV s^−1^ to 500 mV s^−1^. Artemether's direct response to pH indicates possible protonation of artemether or its reaction intermediate during its electrochemical reduction.

Dissolved oxygen was found to distort the resulting cyclic voltammogram from artemether substantially. Sodium sulfite improves signal resolution from artemether in air-equilibrated PBS. There is no difference in mean sensitivity between chronocoulometric quantification of artemether from total charge in nitrogen-equilibrated PBS and 1 mM sodium sulfite in air-equilibrated PBS.

Future work should examine the validity of these methods with substandard and falsified artemether–lumefantrine tablets. These methods should also be tested with other artemisinin derivatives, such as artesunate and dihydroartemisinin, and their artemisinin-based combination therapies. Multiple therapeutic formulations of artemisinin-based combination therapies should be considered, including intravenous, capsule, tablet, and suppository formulations. Finally, these methods should be tested with disposable electrodes and a portable potentiostat to assess feasibility of translation to field-based settings.

## Author contributions

Anna Hildebrand: data curation, formal analysis, investigation, methodology, software, validation, visualization, writing – original draft, writing – review and editing, funding acquisition. Mariam Merchant: investigation, validation, formal analysis. Danny O'Hare: conceptualization, writing – review and editing, funding acquisition, project administration, supervision.

## Conflicts of interest

There are no conflicts to declare.

## Supplementary Material

AY-016-D3AY01837G-s001
