# Supplementary material for: Voltammetric methods for electrochemical characterization and quantification of artemether-based antimalarials
Source: Anal Methods. 2023 Dec 13;16(2):161–9. doi: 10.1039/d3ay01837g (PMC10765968; doi:10.1039/d3ay01837g)
Supplement: AY-016-D3AY01837G-s001 [file AY-016-D3AY01837G-s001.pdf]

Electronic Supplementary Material (ESI) for Analytical Methods.
This journal is © The Royal Society of Chemistry 2023

Electronic Supplementary Information

Table of Contents

1. Introduction 2

2. Materials and methods 3

2.1 Electrochemical cell components 3

2.1 a) Supporting electrolyte selection 3

2.1 b) Solvent Selection 3

2.2 Comparison of pure artemether, filtered Riamet® tablets and unfiltered Riamet® tablets with cyclic voltammetry and chronocoulometry 3

2.2 a) Filter selection 3

3. Results and discussion 5

3.1 Quantification of pure artemether, filtered and unfiltered Riamet® tablets with chronocoulometry 5

3.2 Cyclic voltammetry with artemether and variable scan rates 8

.... 8

3.3 Artemether's response to pH with cyclic voltammetry 9

3.4 Dissolved oxygen effect on artemether with cyclic voltammetry 9

3.5 Sodium sulfite in air-equilibrated PBS effect on artemether with cyclic voltammetry 11

3.6 Quantification of artemether with sodium sulfite in air-equilibrated PBS with chronocoulometry 12

4. References 13

1. Introduction

The expected range in concentration of dissolved oxygen is necessary to determine as signal interference from dissolved oxygen is expected as the reduction potential of oxygen on glassy carbon electrodes (-0.6 V vs. Ag/AgCl [8]) occurs near the reduction potential of artemether (-1.2 V vs. Ag/AgCl) in phosphate buffer with a pH of 7.55. Nevertheless, there is a large overvoltage for the reduction of oxygen, therefore potentials significantly more negative than E_0 for oxygen are required for measurable analyte current [9].

The concentration of dissolved oxygen can be predicted with the Clausius-Clapeyron equation, which describes the relationship between temperature and vapor pressure of gasses [10]:

$$1)\quad \ln\left(\frac{P_1}{P_2}\right) = \frac{\Delta H_{vap}}{R}\left(\frac{1}{T_2} - \frac{1}{T_1}\right)$$

Where ΔH_{vap} is the enthalpy of vaporization of the gas ($J\ mol^{-1}$).

Henry's Law constant (k_H in *M/atm*) relates the partial pressure of a species in the gas phase (p_g in atm) with the concentration of that species in the aqueous phase (c_a in M) [11]:

$$2)\quad k_H \stackrel{\text{def}}{=} c_a/p_g$$

Combining Henry's Law constant with the Clausius-Clayeron equation yields the van't Hoff equation [11]:

$$3)\quad k_H = k_H^{\ominus} * \exp\left(\frac{-\Delta_{soln}H}{R}\left(\frac{1}{T} - \frac{1}{T^{\ominus}}\right)\right)$$

Where $k_H^{\ominus}$ represents Henry's Law constant under standard conditions ($T^{\ominus}$ = 298.15 K).

The temperature dependance of dissolved oxygen can be explained by Henry's Law as a function of temperature[11]:

$$4)\quad \frac{-d\ln k_H}{d\left(\frac{1}{T}\right)} = \frac{-\Delta_{soln}H}{R} = 1700\ K$$

The partial pressure of oxygen can be calculated with the following relationships[12]:

$$5)\quad p_{DryAir} = p_{total} - p_{H_2O}$$

$$6)\quad p_{O_2} = 0.2095 * \left(p_{Total} - p_{H_2O}\right)$$

The partial pressure of oxygen will vary based on temperature, altitude, and relative humidity. Assuming high altitude (3,000 m), high temperature (50°C) and 100% humidity, $pH2O$ is 12,300 Pa and $ptotal$ is 70,108 Pa[13]. Using the above equations, the concentration of dissolved oxygen is calculated as 92 µM. On the other hand, at sea level with a low temperature (5°C), $pH2O$ is defined as 860 Pa[14], so the concentration of dissolved oxygen is calculated as 375 µM. Thus, the concentration of dissolved oxygen will vary from 92 µM to 375 µM.

2. Materials and methods

2.1 Electrochemical cell components

2.1 a) Supporting electrolyte selection

Phosphate-buffered saline (PBS) with a neutral pH was chosen for the supporting electrolyte solution. PBS was selected because of its long-term stability in environments with a high ambient temperature (above 40°C) and humidity [15].

2.1 b) Solvent Selection

Dimethyl sulfoxide (DMSO) was selected as the solvent as it maximizes the signal from the API in the presence of excipients.

Literature states the first step of the API recovery process is to dissolve the drug in a solvent which is a good solvent for the API and a poor solvent for as many excipients as possible [16]. This will allow the API to dissolve in the solvent while the excipients are isolated as solids. This solid—liquid extraction helps to purify the API from the excipients when the drug is filtered.

Artemisinin and its derivatives are reported to be sparingly soluble in aqueous buffers, and literature recommends that the artemisinin derivative is first dissolved in a solvent before dilution in the buffer [17, 18]. For the solvent to be accessible for low-resource settings, the solvent must be non-toxic, not require refrigeration and be readily available in target markets.

Although dimethylformamide and methanol are good solvents for artemether [19], they are not ideal for field use due to their known toxicity. Ethanol and dimethyl sulfoxide (DMSO) both meet the defined criteria and are potential solvents for artemether [19, 20]. Zhang et al. reports successful dissolution of the artemisinin with ethanol [21]. Ethanol is a slightly better solvent for solid—liquid extraction of artemether from excipients than DMSO, as it is a poor solvent for 5/7 and 4/7 excipients respectively [22].

Artemether was found to be more stable in ethanol (degradation after 60 minutes) than in DMSO (degradation after 30 minutes). While ethanol showed promising results with pure artemether, DMSO was ultimately selected as the signal from Riamet® dissolved with ethanol was poor. This could be due to ethanol's inability to break down particles of Riamet® tablets and free the API into solution.

The concentration of the API/DMSO stock solution was 16.757 mM. This concentration was chosen because lower concentrations (8.379 mM) prevented the larger Riamet® particles from breaking down, while higher concentrations (33.515 mM) caused artemether to decay quickly, both of which resulted in poor signal from artemether.

2.2 Comparison of pure artemether, filtered Riamet® tablets and unfiltered Riamet® tablets with cyclic voltammetry and chronocoulometry

An AM-LUM formulation was chosen because it is the most widely prescribed ACT in Sub-Saharan Africa [23], as well as the availability of Riamet® in the UK [24].

2.2 a) Filter selection

A 0.22 μM Millipore filter was selected based on the particle size of the excipients and the signal generated from the filtered drug solution. The ideal filter selected for API extraction is a filter with a pore size smaller than the excipients but larger than the analyte [16]. The particle size for the target analyte, artemether, is

1.14 µM. Artemether particles are smaller than six out of the seven excipients found in Riamet® tablets. Therefore, ideal pore size should fall within the range of 2 µM to 60 µM.

Ingredient		Particle Size (diameter)	Filter 22uM
API (analyte)	Artemether	1143 ± 22.67 nm	no
API (partner)	Lumefantrine	2551 ± 27.3 nm	no
Excipient	Polysorbate 80	1.6 uM	no
	Hypromellose	50 uM	yes
	Microcrystalline cellulose	< 20uM	maybe
	Colloidal anhydrous silica	30-100 nm	no
	Croscarmellose sodium	25-55 uM	yes
	Magnesium stearate	11 uM	no

Table S1 List of excipients in Riamet® tablets[1-7]

3. Results and discussion

3.1 Quantification of pure artemether, filtered and unfiltered Riamet® tablets with chronocoulometry

Linear Regression Model Statistics							
**Total Charge**				**Anson Slope**			
**Artemether**				**Artemether**			
	Estimate	SE	p-value		Estimate	SE	p-value
Intercept	-3.48E-07	2.88E-07	0.31233	Intercept	-3.81E-07	3.13E-07	0.31093
Slope	2.31E-05	1.29E-06	0.00037619	Slope	2.52E-05	1.40E-06	0.00037368
Number of observations	5			Number of observations	5		
Error degrees of freedom	3			Error degrees of freedom	3		
Root Mean Squared Error	2.76E-07			Root Mean Squared Error	3.01E-07		
R-squared	0.991			R-squared	0.991		
Adjusted R-squared	0.988			Adjusted R-squared	0.988		
F-statistic vs. constant model	323			F-statistic vs. constant model	324		
p-value	0.000376			p-value	0.000374		
**Filtered Riamet® Tablets**				**Filtered Riamet® Tablets**			
	Estimate	SE	p-value		Estimate	SE	p-value
Intercept	7.61E-07	2.94E-07	0.081285	Intercept	8.39E-07	3.21E-07	0.079279
Slope	1.05E-05	1.31E-06	0.00408	Slope	1.14E-05	1.43E-06	0.0041265
Number of observations	5			Number of observations	5		
Error degrees of freedom	3			Error degrees of freedom	3		
Root Mean Squared Error	2.83E-07			Root Mean Squared Error	3.08E-07		
R-squared	0.955			R-squared	0.955		
Adjusted R-squared	0.94			Adjusted R-squared	0.94		
F-statistic vs. constant model	64			F-statistic vs. constant model	63.5		
p-value	0.00408			p-value	0.00413		
**Unfiltered Riamet® Tablets**				**Unfiltered Riamet® Tablets**			
	Estimate	SE	p-value		Estimate	SE	p-value
Intercept	1.98E-07	3.32E-07	0.59204	Intercept	2.14E-07	3.60E-07	0.5937
Slope	1.61E-05	1.48E-06	0.0016772	Slope	1.76E-05	1.61E-06	0.001653
Number of observations	5			Number of observations	5		
Error degrees of freedom	3			Error degrees of freedom	3		
Root Mean Squared Error	3.19E-07			Root Mean Squared Error	3.46E-07		
R-squared	0.975			R-squared	0.975		
Adjusted R-squared	0.967			Adjusted R-squared	0.967		
F-statistic vs. constant model	118			F-statistic vs. constant model	119		
p-value	0.00168			p-value	0.00165		

Table S2 Linear regression statistics from dynamic range for artemether quantification with total charge and Anson slope.

Variability between Artemether, Filtered Riamet® and Unfiltered Riamet® for Each Quantification Method				
**Total Charge**			**Anson Slope**	
Intercept			Intercept	
Within sample variance (n = 15)	4.65E-13		Within sample variance (n = 15)	5.51E-13
Between sample variance (n = 3)	3.08E-13		Between sample variance (n = 3)	3.72E-13
F-statistic	0.6611		F-statistic	0.675
Slope			Slope	
Within sample variance (n = 15)	9.29E-12		Within sample variance (n = 15)	1.10E-11
Between sample variance (n = 3)	1.98E-11		Between sample variance (n = 3)	4.76E-11
F-statistic	2.1356		F-statistic	4.3273

Table S3 Comparison of variability for intercept (adsorption) and slope (sensitivity) for artemether, filtered Riamet® and unfiltered Riamet® for quantification with total charge and Anson slope.

Pairwise Comparison of Slope Means for Each Quantification Method				Pairwise Comparison of Intercept Means for Each Quantification Method		
**Total Charge**				**Total Charge**		
**Artemether + Filtered Riamet® (n = 5)**	T-statistic	6.8402		**Artemether + Filtered Riamet® (n = 5)**	T-statistic	2.6969
	P-value	6.61E-05			P-value	1.36E-02
**Artemether + Unfltered Riamet® (n = 5)**	T-statistic	3.572		**Artemether + Unfltered Riamet® (n = 5)**	T-statistic	1.2455
	P-value	0.0036			P-value	0.1241
**Filtered Riamet® + Unfiltered Riamet® (n = 5)**	T-statistic	2.8093		**Filtered Riamet® + Unfiltered Riamet® (n = 5)**	T-statistic	1.2689
	P-value	0.0114			P-value	0.1201
**Anson Slope**				**Anson Slope**		
**Artemether + Filtered Riamet® (n = 5)**	T-statistic	6.8769		**Artemether + Filtered Riamet® (n = 5)**	T-statistic	2.7213
	P-value	6.37E-05			P-value	0.0131
**Artemether + Unfltered Riamet® (n = 5)**	T-statistic	3.5851		**Artemether + Unfltered Riamet® (n = 5)**	T-statistic	1.2469
	P-value	0.0036			P-value	0.1239
**Filtered Riamet® + Unfiltered Riamet® (n = 5)**	T-statistic	2.843		**Filtered Riamet® + Unfiltered Riamet® (n = 5)**	T-statistic	1.2951
	P-value	0.0109			P-value	0.1157

Table S4 Comparison of means for intercept (adsorption) and slope (sensitivity) for artemether, filtered Riamet® and unfiltered Riamet® for quantification with total charge and Anson slope.

Pairwise Comparsion of Variances for Quantification Methods	
**Artemether**	
Total charge variance (n = 5)	1.47E-01
Anson slope variance (n = 5)	1.46E-01
F-statistic	1.0037
**Filtered Riamet® Tablets**	
Total charge variance (n = 5)	5.29E-01
Anson slope variance (n = 5)	5.31E-01
F-statistic	1.0031
**Unfiltered Riamet® Tablets**	
Total charge variance (n = 5)	3.65E-01
Anson slope variance (n = 5)	3.61E-01
F-statistic	1.0097

Pairwise Comparison of Means for Quantification Methods	
**Artemether**	
T-statistic (n = 5)	3.13E-04
P-value	0.4999
**Filtered Riamet® Tablets**	
T-statistic (n = 5)	0.0052
P-value	0.498
**Unfiltered Riamet® Tablets**	
T-statistic (n = 5)	9.01E-04
P-value	0.4997

Table S5 & S6 Pairwise comparisons with each sample type with normalized linear regression models for total charge and Anson slope. Regression models were normalized by dividing each group by its maximum value. (Left) Comparison of variances (Right) Comparison of means

3.2 Cyclic voltammetry with artemether and variable scan rates

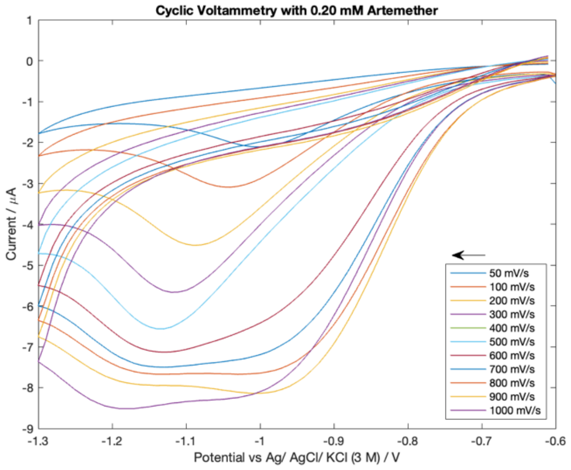

Fig. S1 Raw cyclic voltammetry scans from 0.20 mM artemether in nitrogen-equilibrated PBS (pH 7.54) with scan rates varying from 50 mV/s to 1000 mV/s

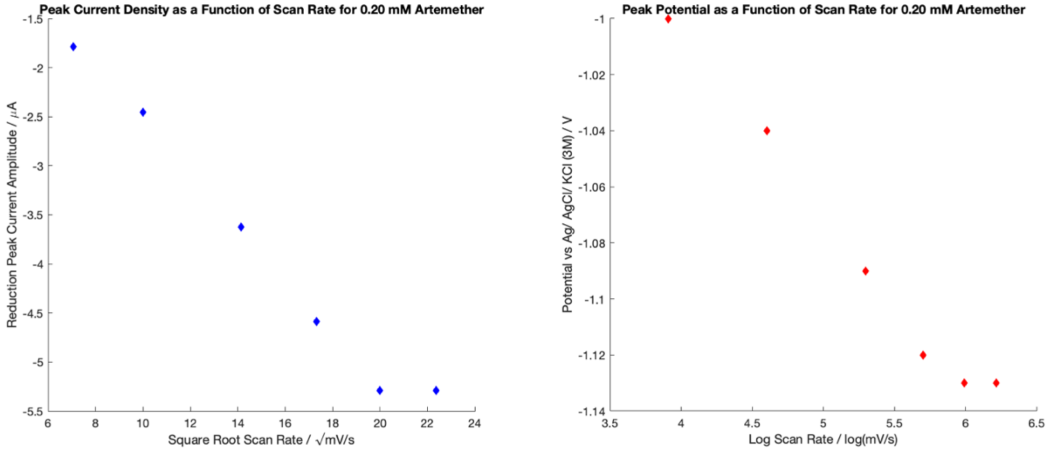

Fig. S2 Reduction peak current density and reduction peak potential extracted from **Fig. S1** with scan rates from 50 mV/s to 500 mV/s. (left) Peak current density as a function of square root scan rate. (right) peak potential as a function of log scan rate.

3.3 Artemether's response to pH with cyclic voltammetry

$$1) \quad ART + 2e^- + 2H^+ \rightleftharpoons products$$

$$2) \quad E = E_0 - \frac{RT}{nF}\ln\left(\frac{[pdts]}{[ART][H^+]^2}\right)$$

$$3) \quad E = E_0 - \frac{RT}{nF}\ln\left(\frac{[pdts]}{[ART]}\right) - \frac{RT}{nF}(2\ln[H^+])$$

$$4) \quad E = E_0 - \frac{RT}{nF}\ln\left(\frac{[pdts]}{[ART]}\right) + \frac{RT}{nF}(2 * 2.303\log[H^+])$$

$$5) \quad E = E_0 - \frac{RT}{nF}\ln\left(\frac{[pdts]}{[ART]}\right) + \frac{RT}{nF}(2 * 2.303 * pH)$$

$$6) \quad E = E_0 - \frac{RT}{nF}\ln\left(\frac{[pdts]}{[ART]}\right) + 59.159mV * pH$$

Derivation from Nernst equation of expected half-peak potential shift if reduction of artemether is coupled with protonation.

3.4 Dissolved oxygen effect on artemether with cyclic voltammetry

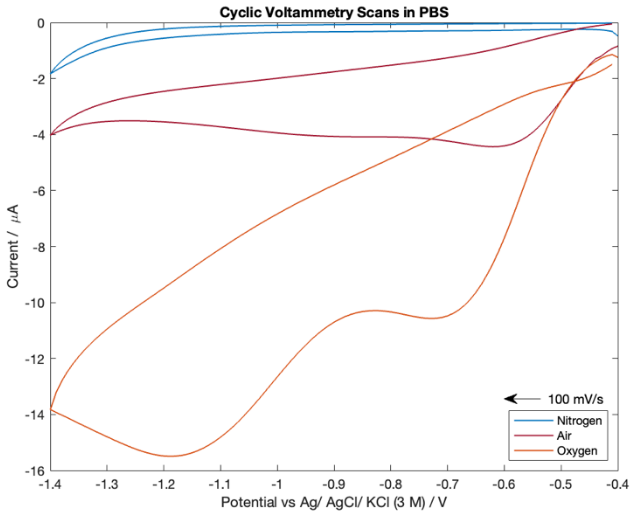

Fig. S3 Blank cyclic voltammetry scans for air-, nitrogen- and oxygen-equilibrated PBS with pH of 7.55

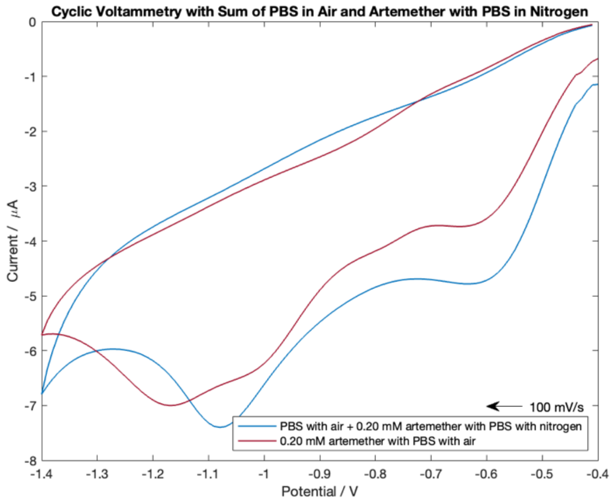

Fig. S4 Additive effect of blank cyclic voltammetry scan with air-equilibrated PBS and 0.20 mM artemether with nitrogen-equilibrated PBS (blue trace). PBS in air-equilibrated PBS with 0.20 mM artemether (red trace). PBS has a pH of 7.55 for scans.

3.5 Sodium sulfite in air-equilibrated PBS effect on artemether with cyclic voltammetry

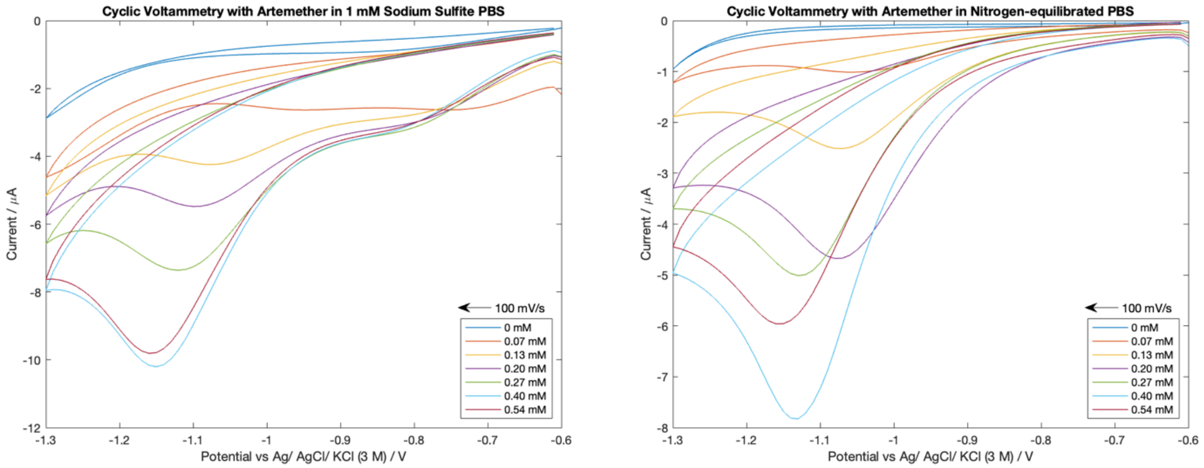

Fig. S5 Cyclic voltammetry scans with standard additions of artemether. (left) 1 mM sodium sulfite in air-equilibrated PBS (pH of 7.55) (right) nitrogen-equilibrated PBS (pH of 7.54)

3.6 Quantification of artemether with sodium sulfite in air-equilibrated PBS with chronocoulometry

Linear Regression Model Statistics								
Nitrogen-equilibrated PBS					1 mM Sodium Sulfite in Air-equilibrated PBS			
	Estimate	SE	p-value			Estimate	SE	p-value
Intercept	-8.45E-07	4.34E-07	0.14648		Intercept	-1.39E-07	6.47E-07	0.84383
Slope	3.43E-05	1.79E-06	0.00030868		Slope	3.29E-05	2.67E-06	0.001147
Number of observations	5				Number of observations	5		
Error degrees of freedom	3				Error degrees of freedom	3		
Root Mean Squared Error	4.58E-07				Root Mean Squared Error	6.83E-07		
R-squared	0.992				R-squared	0.981		
Adjusted R-squared	0.989				Adjusted R-squared	0.974		
F-statistic vs. constant model	369				F-statistic vs. constant model	152		
p-value	0.000309				p-value	0.00115		

Table S7 Linear regression statistics from artemether quantification with total charge with nitrogen-equilibrated PBS (pH of 7.54) and 1 mM sodium sulfite in air-equilibrated PBS (pH of 7.55).

Variability between Artemether Quantification in Nitrogen-equilibrated PBS and 1 mM Sodium Sulfite in Air-equilibrated PBS	
Intercept	
Within sample variance (n = 10)	1.52E-12
Between sample variance (n = 2)	6.23E-14
F-statistic	0.0411
Slope	
Within sample variance (n = 10)	2.57E-11
Between sample variance (n = 2)	2.46E-13
F-statistic	0.0095

Table S8 Comparison of intercept (adsorption) and slope (sensitivity) for artemether quantification with total charge with nitrogen-equilibrated PBS (pH of 7.54) and 1 mM sodium sulfite in air-equilibrated PBS (pH of 7.55).

4. References

1. P. Shende, P. Desai, R. S. Gaud and R. Dhumatkar, *Artificial Cells, Nanomedicine, and Biotechnology*, 2017, **45**, 1597-1604.
2. S. Borra, Chinnaeswaraiah and G. Kamalakar, *Research Journal of Pharmacy and Technology*, 2018, **11**, 4285.
3. S.-E. Chemical, *Hypromellose, Metolose SR*, Shin-Etsu.
4. Sigma-Aldrich, Cellulose microcrystalline, 1.02331).
5. H. Scientific, Size Analysis of Colloidal Silica).
6. IMCD, Croscarmellose Sodium).
7. Roquette, Roquette Magnesium Stearate HS
High Specific Surface Area).
8. H. Zhang, C. Lin, L. Sepunaru, C. Batchelor-McAuley and R. G. Compton, *Journal of Electroanalytical Chemistry*, 2017, **799**, 53-60.
9. D. O. H. Eleni Bitziou, and Bhavik Anil Patel*, *Anal Chem*, 2009.
10. T. Fujimoto, K. Takeda and T. Nonaka, in *Developments in Surface Contamination and Cleaning (Second Edition)*, eds. R. Kohli and K. L. Mittal, William Andrew Publishing, Oxford, 2008, DOI: https://doi.org/10.1016/B978-0-323-29960-2.00007-1, pp. 197-329.
11. R. Sander, *Compilation of Henry's Law Constants for Inorganic and Organic Species of Potential Importance in Environmental Chemistry*, Air Chemistry Department, Max-Planck Institute of Chemistry, Air Chemistry Department, Max-Planck Institute of Chemistry, 1999.
12. s. Sharma and D. Rawat, 2018.
13. H. Schön, in *Handbook of Purified Gases*, ed. H. Schoen, Springer Berlin Heidelberg, Berlin, Heidelberg, 2015, DOI: 10.1007/978-3-540-32599-4_2, pp. 11-37.
14. B. P. L. A. Findlay, Wiley, Halsted press book, 1972.
15. Sigma-Aldrich, Phosphate Buffered Saline System, https://www.sigmaaldrich.com/deepweb/assets/sigmaaldrich/product/documents/254/699/pbs1dat.pdf, (accessed 02/09/22).
16. D. E. Pratama, W. C. Hsieh, A. Elmaamoun, H. L. Lee and T. Lee, *ACS Omega*, 2020, **5**, 29147-29157.
17. R. Jain and Vikas, *Colloids and Surfaces B: Biointerfaces*, 2011, **88**, 729-733.
18. C. Chemical, Artesunate Product Information, https://cdn.caymanchem.com/cdn/insert/11817.pdf, (accessed 02/09/22).
19. C. Chemical, Artemether, https://cdn.caymanchem.com/cdn/insert/11815.pdf, (accessed 05/09/22).
20. Sigma-Aldrich, Artemether, https://www.sigmaaldrich.com/GB/en/product/sigma/a9361, (accessed 05/09/22).
21. F. Zhang, D. K. Gosser and S. R. Meshnick, *Biochemical Pharmacology*, 1992, **43**, 1805-1809.
22. D. M. Shrikant Pagay, *Journal*.
23. S. D. e. al., *AMS*, 2018.
24. N. P. U. Ltd, Riamet 20 mg/120 mg Tablets, https://www.medicines.org.uk/emc/product/1628/smpc#gref, (accessed 02/09/22).
